# The Impact of Positive Youth Development Attributes and Life Satisfaction on Academic Well-Being: A Longitudinal Mediation Study

**DOI:** 10.3389/fpsyg.2020.02126

**Published:** 2020-09-01

**Authors:** Daniel T. L. Shek, Wenyu Chai

**Affiliations:** Department of Applied Social Sciences, The Hong Kong Polytechnic University, Hong Kong, China

**Keywords:** positive youth development, life satisfaction, academic stress, academic satisfaction, longitudinal study

## Abstract

While research studies revealed that positive youth development (PYD) attributes have beneficial impact on adolescent developmental outcomes, whether and how PYD qualities are related to academic well-being (such as academic stress and academic satisfaction) are unclear. Based on a longitudinal study (*N* = 2,312 secondary school students; Mage = 12.54 ± 0.68; 51% female) in Hong Kong, the present study tested a longitudinal mediation model in which it was hypothesized that PYD qualities predicted life satisfaction, academic stress, and academic satisfaction, with satisfaction with life mediating the influence of PYD qualities on academic well-being. Results showed that PYD qualities positively predicted academic satisfaction but negatively predicted academic stress over time. While life satisfaction partially mediated the influence of PYD attributes on academic satisfaction, it fully mediated the influence of PYD attributes on academic stress. The present study supports the proposed conceptual model and underscores the role of PYD qualities in academic well-being through the mediation of life satisfaction.

## Introduction

During the past two decades, there has been a change in the paradigm of adolescent research. Rather than viewing adolescents as “problems,” the “positive youth development” (PYD) perspective highlights resources, potentials, and strengths in adolescents (Damon, 2004; Shek et al., 2019). For example, Lerner (2005) theorized that PYD qualities could be indicated by “Five Cs,” including “competence, confidence, connection, character, and caring” (p. 31). The “Five Cs” could enable an adolescent to function as “an active agent in one’s own development,” which would increase his/her well-being but decrease his/her problem behavior (Lerner, 2005, p.32). Catalano et al. (2004) put forward 15 indicators of PYD based on an evaluation of positive youth development programs in the United States. The 15 indicators include “bonding,” “resilience,” “social competence,” “emotional competence,” “cognitive competence,” “behavioral competence,” “moral competence,” “self-determination,” “spirituality,” “self-efficacy,” “clear and positive identity,” “belief in the future,” “recognition for positive behavior,” “prosocial involvement,” and “prosocial norms” (Catalano et al., 2004, p.101–102). Benson et al. (2006) argued that PYD could be shaped by both external and internal assets, which contributes to adolescent well-being and later success. Consistent with the PYD theories, research findings showed that PYD qualities were linked to a broad range of adolescent developmental outcomes including internalizing and externalizing behaviors, psychological adjustment as well as prosocial behavior (Eichas et al., 2010; Schwartz et al., 2010; Ciocanel et al., 2017; Shek and Zhu, 2018; Zhou et al., 2020). However, few studies have addressed the mediating role of life satisfaction in the influence of PYD qualities on adolescent developmental outcomes, particularly in different Chinese societies (Sun and Shek, 2010, 2012). Studies on the effect of PYD qualities on academic well-being (such as academic stress and academic satisfaction) are also scarce in the international literature (Shek and Wu, 2016).

As a commonly used index of subjective well-being, life satisfaction is defined as a person’s overall cognitive evaluation of the quality of own life (Gilman and Huebner, 2003). Studies showed that life satisfaction was influenced by both environmental and individual factors (e.g., McKnight et al., 2002), including PYD qualities such as emotional intelligence, self-esteem, self-efficacy, social competence, spirituality, and character strengths (Huebner et al., 2004; Proctor et al., 2009; Rey et al., 2011; Howell et al., 2013; Chen et al., 2017). In addition, cross-sectional and longitudinal studies also revealed that PYD qualities positively predicted satisfaction with life among adolescents (Sun and Shek, 2010, 2013; Mohamad et al., 2014). Theoretically, PYD qualities such as self-efficacy, self-worth, and good social relationships (Lerner et al., 2009) would lead to one’s positive appraisal of one’s life (i.e., higher life satisfaction). As a result, it is commonly hypothesized that PYD qualities are a positive predictor of satisfaction with life (Sun and Shek, 2010, 2012).

Although studies suggest that PYD qualities and related intervention could promote academic performance of students (Durlak et al., 2010; Yu et al., 2018), it is not clear about the relationships between PYD qualities and academic well-being such as academic satisfaction and academic stress. According to Marchiondo et al. (2010), academic satisfaction was defined as “the attraction or positive feelings that a student associates with the college or program in question” (p. 610). Jadidian and Duffy (2012) also proposed that academic satisfaction mainly covers one’s satisfaction with academic programs and classes. Therefore, students’ satisfaction with their curriculum is an essential component of their academic satisfaction. However, academic satisfaction is different from academic engagement with the latter referring to students’ devotion and involvement in their academic study such as participating in classes on time, preparation for classwork, and efforts in doing homework (Singh et al., 2002). Students’ academic satisfaction is a major concern of educators as it was regarded as one important “subjective indicator” of an individual’s success in the academic domain (Wach et al., 2016, p.1). Academic satisfaction also reflects how successfully a program is conducted (Albarrak et al., 2013), which is closely related to student retention (Wilkins-Yel et al., 2018). A few studies revealed that PYD qualities positively predicted academic satisfaction, including self-efficacy, emotional competence, and conscientiousness (Lent et al., 2007; van Schaick et al., 2007; Sheu et al., 2016). Also, research studies revealed that PYD attributes such as hope and optimism predicted school satisfaction (Wilkins et al., 2014; Sun, 2016). The common conjecture generated from the scientific literature is that PYD qualities are a positive predictor of academic satisfaction.

Besides academic satisfaction, academic stress is also an important factor influencing adolescents’ psychosocial development, including school adjustment. While there are different views on academic stress, one common conception is that academic stress is students’ “subjective experience of distress” under certain academic-related stimulus or stressors (Putwain, 2007). Perceived academic stress might come from different stressors produced by one’s academic study such as high workload, meeting tight deadlines, and handling multiple tasks (Rayle and Chung, 2007; Bedewy and Gabriel, 2015). Particularly, research suggests that “academic work and its related assessments” are major stressors for students in secondary schools (Putwain, 2007). Therefore, highly pressured academic study and academic programs could be the major stressors or stimuli leading to academic stress. While a similar concept related to academic stress is academic burnout, the two concepts are different. Scholars conceptualized academic burnout to be a psychological reaction or syndrome that is shaped by academic stress (Shin et al., 2012; Charkhabi et al., 2013). Empirical research also showed that academic stress was an important predictor of academic burnout (Lin and Huang, 2013). Academic stress is important to students because it is related to a wide range of physical, mental, and academically related problems such as depression, low motivation to learn, and physical illness (Ang and Huan, 2006; Hystad et al., 2009; Liu, 2015). Unfortunately, the extant literature mainly focuses on environmental predictors and outcomes of academic stress, with few studies examining personal predictors of perceived academic stress. Some limited studies revealed that perceived academic stress was negatively predicted by psychological strengths such as grit, optimism, and emotional intelligence (Huan et al., 2006; Bao et al., 2015; Lee, 2017) as well as cognitive abilities (Giota and Gustafsson, 2017). Taking together, the literature suggests a negative influence of PYD attributes on perceived academic stress.

Considering the relationship between life satisfaction and academic satisfaction, the proposal that life satisfaction influences academic satisfaction is supported by the “top-down model” of life satisfaction. In the “top-down model,” global satisfaction with life is regarded as “a global propensity to experience things in positive ways” which “influences the momentary interactions an individual has with the world” (Diener, 1984, p.565). In other words, life satisfaction is not the sum of satisfaction with different aspects of life, but more a relatively stable psychological characteristic that influences individuals’ perceptions of, and reaction to, their environment (Diener, 1984; Schimmack et al., 2002). On the basis of the “top-down model,” it is hypothesized that life satisfaction would positively contribute to academic satisfaction. Some studies showed that higher life satisfaction predicted higher job and career satisfaction among adolescents (Judge and Watanabe, 1993; Diener and Tay, 2017). In addition, research showed that life satisfaction led to higher student motivation and school engagement (Lewis et al., 2011; Dilling, 2016), which predicted increased academic satisfaction (Wach et al., 2016; Johnson et al., 2017). However, few studies have further explored such findings using longitudinal research designs.

While perceived stress is commonly conceived as a precursor of life satisfaction, some research studies suggest that life satisfaction might also be an antecedent of perceived stress. For example, two studies showed that global life satisfaction was an important predictor of perceived stress in university students (Sheets et al., 1993; Saleh et al., 2017). Besides, recent longitudinal research on young ex-offenders showed that life satisfaction negatively predicted later perceived stress, but early perceived stress did not predict later life satisfaction (Tang and Chan, 2017). Another study also found that perceived stress was a mediator on the influence of global life satisfaction on post-trauma physical and psychological health (Tremblay et al., 2006). Taken together, these studies indicate that as a psychological strength, life satisfaction could also help an individual to reduce his or her perceived level of stress. Therefore, it can be asserted that life satisfaction predicts perceived academic stress over time.

Existing literature suggests that life satisfaction is not only a predictor of adolescent outcomes, but also a mediator of the influence of other personal and environmental factors on adolescent outcomes (e.g., Otero-López et al., 2011; Yamawaki et al., 2011). Particularly, three research studies revealed that satisfaction with life functioned as a mediator in the impact of PYD qualities on problem behaviors among Chinese youth in Hong Kong (Sun and Shek, 2010, 2012, 2013). Another research also revealed that life satisfaction mediated the influence of recognition for positive behavior (an indicator of PYD) on personal growth initiatives among university students (Stevic and Ward, 2008). Furthermore, Lambert et al. (2009) revealed that satisfaction with life mediated the predictive effect of gratitude on materialism. These studies suggest the mediating role of life satisfaction in the influence of PYD qualities on other adolescent developmental outcomes, such as academic satisfaction and perceived academic stress.

The existing literature in this field shows several limitations. First, while there are studies on PYD qualities and adolescent outcomes such as problem behavior, the predictive role of PYD qualities in adolescent academic well-being indexed by academic satisfaction and academic stress has not been systematically investigated. Second, while studies revealed that both PYD qualities and life satisfaction were linked to adolescent outcomes, whether satisfaction with life has a mediating effect on the influence of PYD qualities on adolescent outcomes is unclear. As studies showed the mediating effect of life satisfaction on the influence of PYD qualities on other adolescent outcomes such as problem behavior (Sun and Shek, 2010, 2012), it is interesting to investigate the mediating effect of life satisfaction on the impact of PYD qualities on academic well-being. Third, while adolescents’ academic development is an important research area, most of the extant studies mainly focused on academic performance and achievement, with few studies investigating academic satisfaction and stress (i.e., academic well-being) as outcomes. Fourth, the existing studies on PYD qualities and life satisfaction mainly adopted cross-sectional research designs, with very few longitudinal studies. Finally, as most of the existing PYD studies were performed in Western contexts, research on PYD qualities and adolescent outcomes in non-Western contexts such as Chinese cultures is strongly needed.

Research in this area would be particularly meaningful in the context of Hong Kong, which inherits the Chinese cultural tradition of morbid emphasis on academic excellence. At the same time, Hong Kong has also experienced a significant and systematic educational reform in the past decade. Under strong Chinese cultural influence, Hong Kong families and parents still strongly emphasize academic excellence in their children, which might cause high academic stress among Hong Kong adolescents. Adding to this situation is a structural change of the secondary school curriculum in Hong Kong since 2009. A “New Secondary School Curriculum” has been implemented that aims at building a more flexible learning system, promoting all-around development of students and developing their life-long skills for the 21st century (Wong, 2011; Cheung and Jhaveri, 2016). The new curriculum has brought about significant change in secondary school students’ study: it shortens students’ senior secondary study from four years to three years, changes students’ examination requirements, and incorporates a compulsory new interdisciplinary subject named “Liberal Studies” (Shek and Leung, 2014; Tan, 2019). Although the intention is good, the new curriculum might increase students’ academic stress. Not only do the shortened study years cause stress for students in their preparation for university entrance examinations, but they also increase pressure for students who are not familiar with the subject and how it is taught (Shek and Li, 2016; Tan, 2019). Therefore, it is important and meaningful to identify personal strengths such as PYD qualities and satisfaction with life that protect Hong Kong students from the development of higher academic stress and promote students’ academic satisfaction under the context of educational transformation.

**FIGURE 1:**
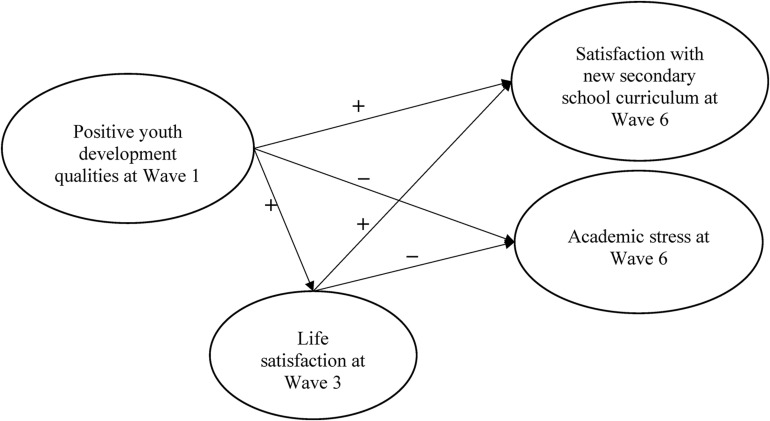
The hypothesized model (life satisfication is hypothesized as a mediator on the relationship between positive youth development qualities to academic satisfication and academic stress). “+” sign indicates positive direction; “-“indicates negative direction.

Adopting a longitudinal research design, the present study attempted to investigate the influence of PYD qualities and life satisfaction on academic satisfaction and academic stress, with life satisfaction hypothesized to be a mediator of the influence of PYD qualities on academic satisfaction and academic stress. This study utilized data from a six-year longitudinal project on the “positive youth development” of Hong Kong adolescents. Three waves of data, i.e., Wave 1, 3, and 6, were used in this study to test the longitudinal mediating effect as the three waves have relatively equal time span. Figure 1 shows the hypothesized mediation model. Based on the above discussion, the following seven hypotheses were formed:

• PYD qualities at Wave 1 would positively predict academic satisfaction at Wave 6 (hypothesis 1a) but negatively predict academic stress at Wave 6 (hypothesis 1b).
• PYD qualities at Wave 1 would positively predict life satisfaction at Wave 3 (hypothesis 2a) and life satisfaction at Wave 3 would positively predict academic satisfaction at Wave 6 (hypothesis 2b) but negatively predict academic stress at Wave 6 (hypothesis 2c).
• Life satisfaction at Wave 3 would mediate the influence of PYD qualities at Wave 1 on academic satisfaction (hypothesis 3a) and academic stress at Wave 6 (hypothesis 3b).

## Materials and Methods

### Participants and Procedure

The data of this study were derived from in a large-scale project on adolescents’ positive development and its precursors as well as outcomes. The project was conducted from the 2009/2010 to 2015/2016 school years, which involved 28 randomly selected Hong Kong secondary schools. Since the 2009/2010 school year, seventh-grade students in these schools had been invited to fill out a paper and pencil questionnaire each year. Before the start of the study, formal written consent was obtained from all participating schools, students, and parents. The participating students were fully informed about the purpose of the study and principles of confidentiality.

The present study used the data collected in Waves 1, 3, and 6. The time span between Wave 1 and Wave 3 is two years (i.e., 24 months), and the time span between Wave 3 and Wave 6 is two years and 10 months (i.e., 34 months). The number of students who completed the three waves of the questionnaire was 2,312. The mean age and educational level of the participants in different waves are shown in Table 1. At Wave 1, the participants reported a mean age of 12.54 ± 0.68 years old and 51% of the participants were female students.

**TABLE 1:** Mean age and grade of the participants in different waves.

Wave	Grade of the participants	Age of the participants Mean (SD)
Wave 1	7	12.54 (0.68)
Wave 3	9	14.53 (0.66)
Wave 6	12	17.20 (0.59)

### Measures

#### Positive Youth Development (PYD) Attributes

Positive Youth Development was assessed using a shortened version of the “Chinese Positive Youth Development Scale” (CPYDS) (Shek et al., 2007). The original version of the scale contains 90 items assessing 15 attributes of positive development: “Bonding,” “Resilience,” “Social Competence,” “Recognition for Positive Behavior,” “Emotional Competence,” “Cognitive Competence,” “Behavioral Competence,” “Moral Competence,” “Self-Determination,” “Self-Efficacy,” “Clear and Positive Identity,” “Beliefs in the Future,” “Prosocial Norms,” and “Spirituality.” In a validation study based on 5,649 Hong Kong secondary school students (Shek and Ma, 2010), the CPYDS showed stable factor structure. In another validation study based on 322 adolescents in Hong Kong (Shek et al., 2007), the scale showed acceptable reliability and criterion-related validity.

The trimmed CPYDS was based on selecting the items with higher factor loadings from each subscale. It contains 44 items measuring the 15 attributes. Some sample items are (1) “When I need help, I believe my father/mother (or guardian) will definitely help me” (“Bonding”); (2) “When facing difficulties, I do not give up easily” (“Resilience”); (3) “I can get along with other people” (“Social Competence”); and (4) “If I’m not happy, I can express my emotions properly” (“Emotional Competence”). Except for “Spiritualty,” all items were rated on a six-point scale (1 = “Strongly Disagree” and 6 = “Strongly Agree”). The items in the subscale of “Spirituality” were rated on a seven-point scale (1 = “my life is very boring/empty” and 7 = “my life is full of energy/excitement”). A higher level of positive development is represented by a higher composite score. To test the internal structure of the trimmed CPYDS, confirmatory factor analyses (CFA) were performed. The resulting global model fit indices are: χ^2^(795, *N* = 2,312) = 4137.106, *p* < 0.001, CFI = 0.91; TLI = 0.89; SRMR = 0.04; RMSEA = 0.043 with 90% CI [0.041, 0.044]. We also tested the local fit of the model by examining the correlation residuals. Based on Kline’s (2016) suggestion, with reference to the 1,034 correlation residuals, only 39 (4%) had absolute values greater than 0.10. The Cronbach alpha for all subscales of the trimmed CPYDS in Wave 1 ranged from 0.70 to 0.88, except for the “Self-Efficacy” subscale with a value of 0.62. The omega coefficients of all subscales also ranged from 0.70 to 0.88, except for the “Self-Efficacy” subscale which cannot yield an omega coefficient because there are only two items. All the subscales also showed acceptable mean inter-item and item-total coefficients (Table 2).

**TABLE 2:** Cronbach’s alpha, omega coefficient, mean inter-item correlation, and mean item-total correlation of different scales and subscales.

	Cronbach’s alpha	Omega coefficient	Mean inter- item correlation	Mean item- total correlation
**Chinese Positive Youth Development Scale (CPYDS)**				
Bonding (BO, 3 items)	0.75	0.77	0.50	0.58
Resilience (RE, 3 items)	0.78	0.79	0.54	0.62
Social Competence (SC, 3 items)	0.85	0.85	0.65	0.71
Recognition for Positive Behavior (PB, 3 items)	0.76	0.76	0.51	0.59
Emotional Competence (EC, 3 items)	0.73	0.75	0.48	0.56
Cognitive Competence (CC, 3 items)	0.80	0.80	0.57	0.65
Behavioral Competence (BC, 3 items)	0.70	0.70	0.44	0.52
Moral Competence (MC, 3 items)	0.72	0.72	0.47	0.54
Self-Determination (SD, 3 items)	0.74	0.76	0.49	0.57
Self-Efficacy (SE, 2 items)	0.62	NA	0.45	0.45
Clear and Positive Identity (SI, 3 items)	0.77	0.77	0.53	0.61
Beliefs in the Future (BF, 3 items)	0.85	0.85	0.65	0.72
Prosocial Involvement (PI, 3 items)	0.79	0.81	0.55	0.64
Prosocial Norms (PN, 3 items)	0.70	0.72	0.44	0.52
Spirituality (SP, 3 items)	0.88	0.88	0.71	0.77
Life Satisfaction (LS, 5 items)	0.86	0.86	0.58	0.69
Academic Stress (AS, 2 items)	0.92	NA	0.86	0.86
**Satisfaction with New Secondary School Curriculum (PNSC)**				
Fondness for and interest in junior curriculum (4 items)	0.95	0.95	0.83	0.88
Perceived benefits of junior curriculum (5 items)	0.95	0.95	0.80	0.86
Fondness for and interest in senior curriculum (4 items)	0.94	0.94	0.81	0.86
Perceived benefits of senior curriculum (5 items)	0.95	0.95	0.78	0.86

*NA: omega coefficients cannot be calculated for a scale with 2 items.*

#### Life Satisfaction (LS)

We used the “Satisfaction with Life Scale” (SWLS) (Diener et al., 1985) to assess the participants’ global evaluation of life satisfaction. The scale comprises five items gauging global life satisfaction of individuals, applicable in different age groups. One sample item is “In most ways my life is close to my ideal.” The five items were answered on a six-point scale (1 = “Strongly Disagree” and 6 = “Strongly Agree”). A higher level of LS is represented by a higher total score. In several studies, Cronbach’s alpha of SWLS ranged from 0.77 to 0.90 (Gouveia et al., 2009; Rosengren et al., 2015). Also, in several validation studies, SWLS showed convergent validity, criterion-based validity, and test-retest reliability (Pavot et al., 1991; Gouveia et al., 2009; Rosengren et al., 2015). The present study used data of LS in Wave 3 as the mediator. Both Cronbach’s alpha and omega coefficients of SWLS in Wave 3 were 0.86 (“mean inter-item correlation” = 0.58; “mean item-total correlation” = 0.69).

#### Academic Stress (AS)

Two items were developed to measure students’ perceived academic stress. One item is “Do you feel pressure in your current studies?” Another item is “Do you feel pressure under the new senior secondary school curriculum?” The first item assesses the students’ subjective experience of distress in their general academic studies as a whole based on the literature on academic stress (Putwain, 2007). The second item assesses students’ subjective experience of pressure or distress specifically toward the new secondary school curriculum which is perceived as a major stressor in students’ academic study. The items were rated on a four-point scale (1 = “Not at all” and 4 = “Very much”). Higher levels of perceived academic stress are indicated by higher scores. The present study used AS in Wave 6 as one outcome variable. Cronbach’s alpha of AS for Wave 6 was 0.92 in the present study.

#### Academic Satisfaction

As literature suggests students’ satisfaction with their academic program is an important aspect of academic satisfaction (Marchiondo et al., 2010; Jadidian and Duffy, 2012) and because the implementation of the “New Secondary School Curriculum” in Hong Kong would have great impact on students’ satisfaction with academics, a measure was developed to gauge students’ academic satisfaction as a function of their positive feelings and perspectives on the whole new secondary school curriculum (PNSC). Originally, PNSC contained a total of 24 items among which six were reverse-coded. Answers to all items were on a six-point scale (1 = “Strongly Disagree” and 6 = “Strongly Agree”), with higher levels of satisfaction indicated by higher scores. As a reliability test showed that the six reverse-coded items had low item-total correlations with other items, the six reverse-coded items were removed from the scale. The removal of reverse-coded items was also based on the argument that negatively worded items would have negative influence on the reliability and validity of the scale, and they may not measure the same construct as positively worded items (Pilotte and Gable, 1990; Dalal and Carter, 2014). The refined PNSC contained 18 items. Conceptually, the 18 items were generated to measure four dimensions: (1) students’ fondness for, and interest in, the junior secondary school curriculum (4 items); (2) students’ perceptions of the benefits of the junior secondary curriculum in promoting positive and holistic development (5 items); (3) students’ fondness for, and interest in, the senior secondary school curriculum (4 items); and (4) students’ perceptions of the benefits of the senior secondary school curriculum in promoting personal development (5 items). The sample items are “I like the new junior/senior high school curriculum” and “The curriculum can enhance my interest in learning.”

On the basis of the new “standards for educational and psychological testing” (American Educational Research Association, 2014), we performed exploratory factor analyses (EFA) and confirmatory factor analyses (CFA) on PNSC to provide evidence for the internal structure of the scale (Kelloway, 1995; Worthington and Whittaker, 2006). The current dataset (*N* = 2,312, Wave 6) was randomly split into two sub-datasets: dataset A (*N* = 1,141) and dataset B (*N* = 1,171). First, we conducted EFA based on dataset A. In the initial round of EFA, “principal axis factors” (PAF) were used as the data did not meet the assumption of a multivariate normal distribution (Costello and Osborne, 2005; Goretzko et al., 2019). Promax rotation was adopted as the rotation method since the hypothesized factors were assumed to be correlated (Brown, 2009). The analyses suggested three factors based on Eigenvalues > 1. However, there were four items highly loaded on two factors, which could not generate a “simple structure,” an important criterion of EFA (Brown, 2009; Watson, 2017). As parallel analyses (PA) (Humphreys and Montanelli, 1975) and “Velicer’s Minimum Average Partial Test” (MAP) (Velicer, 1976) are two other important methods for determining factor numbers, we conducted PA and MAP using SPSS 25 to detect factor numbers. Both analyses suggested four factors should be extracted. Therefore, another round of PAF with Promax rotation was performed by fixing the factor number at four. The analyses yielded a clear and simple four primary factor structure with each item highly loaded on one factor (ranging from 0.55 to 0.99) but not on other factors according to the original conceptual model. The factor loadings are shown in Table 3. The factor correlation matrix showed that the four factors were correlated with each other (*r* = 0.55 to 0.77), justifying the use of Promax rotation. The four factors explained 81.563% of the item total variance. The findings also matched the conceptual model of the scale. Therefore, the four-factor structure of PNSC was adopted for CFA.

**TABLE 3:** Factor loadings of the four factors of PNSC in exploratory factor analyses based on Dataset A (*N* = 1141).

Item	Factor 1	Factor 2	Factor 3	Factor 4
QPJ1	0.05	−0.06	**0.95**	−0.04
QPJ2	0.04	0.13	**0.79**	−0.01
QPJ3	0.03	0.08	**0.88**	−0.01
QPJ4	−0.08	0.07	**0.84**	0.05
QPJ8	−0.05	**0.69**	0.19	0.07
QPJ9	−0.03	**0.82**	0.09	0.03
QPJ10	0.01	**0.99**	−0.03	−0.02
QPJ11	−0.01	**0.95**	0.00	−0.00
QPJ12	0.11	**0.67**	0.10	0.03
QPS1	**0.92**	−0.03	0.05	−0.05
QPS2	**0.65**	−0.06	0.10	0.25
QPS3	**0.94**	−0.01	−0.02	0.02
QPS4	**0.94**	0.06	−0.05	−0.06
QPS8	0.24	0.11	−0.03	**0.60**
QPS9	0.18	0.03	0.01	**0.72**
QPS10	−0.05	−0.04	−0.00	**0.99**
QPS11	−0.03	0.05	0.00	**0.93**
QPS12	0.33	0.03	−0.02	**0.55**

*PNSC = satisfaction with new secondary school curriculum; Factor 1 = students’ fondness for, and interests in junior secondary school curriculum; Factor 2 = students’ perceived benefits from junior secondary curriculum in promoting positive and holistic development; Factor 3 = students’ fondness for, and interest in, senior secondary school curriculum; Factor 4 = students’ perceived benefits from senior secondary school curriculum in promoting personal development. The high factor loadings are bold.*

CFA was conducted on the four-factor model of PNSC based on dataset B. The resulting global model fit indices were: χ^2^(129, *N* = 1,171) = 840.008, *p* < 0.001, CFI = 0.94; TLI = 0.93; SRMR = 0.04; RMSEA = 0.069 with 90% CI [0.064, 0.073]. Based on Hu and Bentler (1999) and Kline (2011, 2016), the CFI value and TLI value were greater than or equal to 0.90, and the RMSEA value below 0.08 suggests adequate model fit. While the chi-square test rejected the model (*p* < 0.001), the SRMR value = 0.04 (<0.08) suggests the approximate fit of the model (Asparouhov and Muthen, 2018). In addition, among 189 correlation residuals, only 5 (3%) had an absolute value > 0.10, thus providing support for the model fit. The factor loadings ranged from 0.84 to 0.96 with the four factors all significantly positively correlated with each other (Table 4). As the four-factor model of PNSC was supported, it was adopted for further analyses in this study. Both Cronbach’s alpha and omega coefficients for the four subscales ranged from 0.94 to 0.95 (“mean inter-item correlation” ranged from 0.78 to 0.83; “mean item-total correlation” ranged from 0.86 to 0.88).

**TABLE 4:** Table 4 Factor loadings of the four factors of PNSC in confirmatory factor analyses based on Dataset B (*N* = 1171).

PNSC	Factor Loading		SE
**Factor 1**			
QPJ1	0.87***		0.02
QPJ2	0.90***		0.01
QPJ3	0.96***		0.01
QPJ4	0.86***		0.02
**Factor 2**			
QPJ8	0.84***		0.02
QPJ9	0.86***		0.02
QPJ10	0.95***		0.01
QPJ11	0.94***		0.01
QPJ12	0.81***		0.02
**Factor 3**			
QPS1	0.91***		0.01
QPS2	0.84***		0.02
QPS3	0.94***		0.01
QPS4	0.89***		0.01
**Factor 4**			
QPS8	0.88***		0.01
QPS9	0.90***		0.01
QPS10	0.92***		0.02
QPS11	0.92***		0.01
QPS12	0.84***		0.02

	**Correlations among different factors**		
	**Factor 1**	**Factor 2**	**Factor 3**

Factor 1	–		
Factor 2	0.78***	–	
Factor 3	0.58***	0.58***	–
Factor 4	0.57***	0.72***	0.78***

PNSC = satisfaction with new secondary school curriculum; Factor 1 = students’ fondness for and interests in junior secondary school curriculum; Factor 2 = students’ perceived benefits from junior secondary curriculum in promoting positive and holistic development; Factor 3 = students’ fondness for, and interest in, senior secondary school curriculum; Factor 4 = students’ perceived benefits from senior secondary school curriculum in promoting personal development. ***p < 0.001.

### Data Analyses

Means, standard deviations, and correlations among different variables were computed first. Second, two steps (Anderson and Gerbing, 1988) were followed to examine the hypothesized mediation model by using Structural Equation Modeling (SEM) and MPLUS 8.1. First, a measurement model was tested for four inter-correlated latent variables, including Positive Youth Development (PYD, indicated by its 15 dimensions), Life Satisfaction (LS, indicated by its five items), Academic Stress (AS, indicated by its two items), and Perspective on the New Secondary School Curriculum (PNSC, indicated by its four factors as sub-latent variables, which were further indicated by their respective items). Second, on the condition of satisfactory model fit of the measurement model, the mediation model was then examined, with LS being hypothesized as the mediator on the relationship between PYD and the two indexes of academic well-being (AS and PNSC).

One assumption of SEM was the multivariate normal distribution of the data (Kaplan, 2008). This assumption was tested by MPLUS. The results showed that *p* < 0.001 for both multivariate skew and kurtosis tests. Therefore, MLR (“robust maximum likelihood method”) was used as the model estimation method as it is a “robust full information ML estimator” which “is not dependent on the assumption of normality,” and it “yields a robust chi-square test of model fit” (Kaplan, 2008, p.88). The MLR estimation was also adopted based on the following justifications. First, although the data of the outcome variables were collected based on a Likert scale, which is ordinal in nature, studies suggested that MLR could be used for ordinal data with five or more response categories (Byrne, 2012; Raykov, 2012; Li, 2016). Second, although there are other estimation methods suitable for ordinal data such as WLSMV, research has shown that the estimations of MLR and WLSMV are quite similar when the sample size is large (Hansson and Gustafsson, 2013) and although MLR is not “specifically developed for use with categorized data, performed surprisingly well” (Bandalos, 2014, p. 116). As the present study had a sample size of over 2,000, we used MLR as the estimation method.

The study used multiple indices to assess the fitness of the models, including CFI (“comparative fit index”), TLI (“Tucker-Lewis index”), RMSEA (“root mean square error of approximation”), and SRMR (“standardized root mean square residual”). Based on Hu and Bentler (1999) and Kline (2011, 2016), a model would be acceptable if the CFI value and TLI value are above 0.90, and the RMSEA value is below 0.08. These criteria have been widely used in many research studies not only for ML but also for MLR estimation (e.g., Boelen et al., 2010; Hill et al., 2015). Also, a model would be approximately well fit with a SRMR value below 0.08 when the chi-square test rejects the model (Asparouhov and Muthen, 2018). In addition, R software (version 3.6.3 with Lavaan) was used to test the local fit of the CFA and SEM models by examining the correlation residuals. According to Kline (2016), the local fit can be established if few (<5%) correlation residuals were smaller than 0.10. Furthermore, bias-corrected bootstrap estimation (95% confidence interval for significance testing) based on 5,000 bootstrap samples was conducted to examine the mediation effects.

## Results

### Descriptive Analyses

Table 5 presents the mean scores and standard deviations of different variables, and mean score correlations between different variables. As predicted, there were significant positive correlations amongst PYD, LS, and PNSC, and these variables were significantly and negatively correlated with AS.

**TABLE 5:** Means, standard deviations, and mean score correlations between different variables.

	Variable	Mean (SD)	1	2	3
1.	Positive Youth Development at Wave 1	4.53 (0.67)	−		
2.	Life Satisfaction at Wave 3	3.78 (1.03)	0.36***	−	
3.	Academic Stress at Wave 6	2.95 (0.74)	−0.06**	−0.11***	−
4.	Satisfaction with New Secondary School Curriculum at Wave 6	3.66 (0.93)	0.27***	0.30***	−0.17***

**p < 0.01; ***p < 0.001.

### Measurement Model

The measurement model comprised four interrelated latent variables (PYD, LS, AS, and PNSC). Results showed adequate model fit: χ^2^(730, *N* = 2,312) = 5666.991, *p* < 0.001, CFI = 0.91; TLI = 0.91; SRMR = 0.05; RMSEA = 0.054 with 90% CI [0.053, 0.055]. The factor loadings ranged from 0.57 to 0.96 (*p* < 0.001), suggesting that the indicators well represented their respective latent variables.

### Analyses of the Mediation Model

**FIGURE 2:**
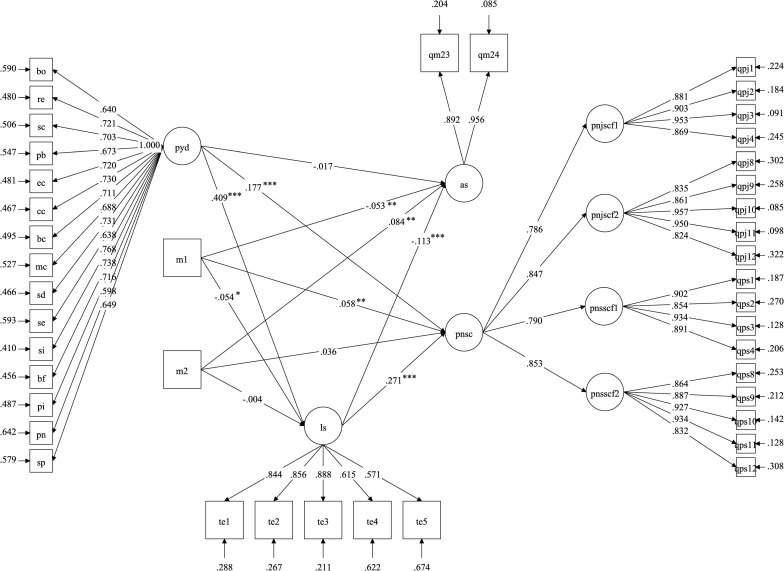
The structural equation model on the mediating effects of life satisfication on the influence of youth development qualities on academic satisfication and academic strees. ***p < 0.001;**p < 0.01;*p < *0.05.* pyd = positive youth development qualities at Wave 1; Is = life satisfication at Wave 3; as = academic strees at Wave 6; pnsc = satisfication with new secondary school curriculam at Wave 6; pnjscf1, pnjscf2, pnsscf1, and pnsscf2 are four factors of pnsc; m1 and m2 are two convariates: m1 = age, m2 = gender. The factors loadings for each latent variable were estimated by fixing one factor loading (unstandardized) to 1.0. The factor loadings shown in figure are standardized factor loadings. The R-square values for the two endogenous variables: as and pnsc are 0.024 and 0 147, respectively.

As the measurement model is acceptable, the mediation model (SEM model) was then examined in which PYD at Wave 1 was hypothesized to predict the two academic well-being variables (PNSC and AS) at Wave 6, with LS at Wave 3 mediating the relationships. Considering that the participants came from 28 schools, we used Type = complex option in MPLUS to control the school influence. Meanwhile, we controlled the age and gender effects in the model. The resulting global model fit indices were: χ^2^(804, *N* = 2,312) = 6058.749, *p* < 0.001, CFI = 0.91; TLI = 0.90, SRMR = 0.05, RMSEA = 0.054 with 90% CI [0.052, 0.055]. Figure 2 shows the model. For local fit, results showed that among the 945 pairs of correlation residuals, 48 (5%) had absolute values over 0.10, which gave marginal support for the local fit of the model. However, when the global fit and local fit findings were taken into account, the evidence provided reasonable support for the proposed model. The estimates of the relationships between different latent variables are presented in Table 6. As shown in Table 6, Wave 1 PYD positively predicted Wave 6 PNSC (total effect: ß = 0.288, *p* < 0.001) but negatively predicted Wave 6 AS (total effect: ß = -0.063, *p* = 0.01). Therefore, Hypotheses 1a and 1b were supported.

**TABLE 6:** Estimated predicting effects of PYD on AS and PNSC with the mediating effect of LS in SEM.

	β	SE
Effect of PYD on LS	0.409***	0.023
Effect of LS on PNSC	0.271***	0.026
Effect of LS on AS	−0.113***	0.025
Total effect of PYD on PNSC	0.288***	0.020
Direct effect of PYD on PNSC	0.177***	0.021
Indirect effect of PYD on PNSC (LS as the mediator)	0.111***	0.013
Total effect of PYD on AS	−0.063^a^	0.025
Direct effect of PYD on AS	–0.017	0.031
Indirect effect of PYD on AS (LS as the mediator)	−0.046***	0.011

PYD = Positive youth development qualities at Wave 1; LS = Life satisfaction at Wave 3; AS = Academic stress at Wave 6; PNSC = Satisfaction with new secondary school curriculum at Wave 6. ^a^*p* = 0.01; ***p < 0.001.

In addition, Wave 1 PYD positively predicted Wave 3 LS (ß = 0.409, *p* < 0.001) and Wave 3 LS positively predicted Wave 6 PNSC (ß = 0.271, *p* < 0.001) but negatively predicted Wave 6 AS (ß = -0.113, *p* < 0.001). Therefore, Hypotheses 2a–2c were supported. Furthermore, Wave 3 LS partially mediated the association between PYD (Wave 1) and PNSC (Wave 6) due to the both significant indirect effect (ß = 0.111, *p* < 0.001) and direct effect (ß = 0.177, *p* < 0.001), which supported Hypothesis 3a. LS (Wave 3) fully mediated the effect of PYD (Wave 1) on AS (Wave 6) based on the significant indirect effect (ß = -0.046, *p* < 0.001) but insignificant direct effect (ß = -0.017, *p* > 0.05). Therefore, Hypothesis 3b was supported. Besides, the estimated correlations among different latent variables were shown in Table 7 where PYD, LS, and PNSC were positively correlated with each other but they were negatively correlated with AS.

**TABLE 7:** Estimated correlations between different latent variables.

	Variable	1	2	3
1.	Positive youth development qualities at Wave 1	−		
2.	Life Satisfaction at Wave 3	0.41***	−	
3.	Academic Stress at Wave 6	−0.06^a^	−0.12***	−
4.	Satisfaction with New Secondary School Curriculum at Wave 6	0.29***	0.34***	−0.20***

^*a*^*p* = 0.01; ***p < 0.001.

To examine the mediating effects further, “bias-corrected bootstrap” estimation based on 5,000 bootstrap samples was also conducted on the mediation model. As shown in Table 8, the 95% confidence intervals for all the two indirect effects with LS as mediator did not cross zero, indicating the significance of these mediation effects.

**TABLE 8:** Results of bias-corrected bootstrap estimation for the mediation model.

	Point estimates	95% CI	
		Lower 2.5%	Upper 2.5%
Effects of PYD on LS	0.409	0.364	0.452
Effects of LS on AS	–0.113	–0.165	–0.067
Effects of LS on PNSC	0.271	0.223	0.324
Total effect of PYD on PNSC	0.288	0.246	0.327
Direct effect of PYD on PNSC	0.177	0.136	0.220
Indirect effect of PYD on PNSC (LS as the mediator)	0.111	0.088	0.139
Total effect of PYD on AS	–0.063	–0.110	–0.014
Direct effect of PYD on AS	–0.017	–0.075	0.044
Indirect effect of PYD on AS (LS as the mediator)	–0.046	–0.070	–0.026

*Bootstrap sampling = 5,000. PYD = Positive youth development qualities at Wave 1; LS = Life satisfaction at Wave 3; AS = Academic stress at Wave 6; PNSC = Satisfaction with new secondary school curriculum at Wave 6.*

## Discussion

This study investigated the predictive effects of PYD qualities and life satisfaction on academic well-being indicated by academic satisfaction and academic stress among Hong Kong adolescents. The study is significant for two reasons. First, as few studies have examined the mediating effect of global life satisfaction on the influence of PYD qualities on adolescent developmental outcomes, this study is an interesting addition to the literature. Second, we examined academic satisfaction and academic stress, which are two important aspects of adolescents’ academic well-being (Trógolo and Medrano, 2012; Wach et al., 2016). As existing research on academic satisfaction and stress focused more on the environmental influences (e.g., Tessema et al., 2012) with relative negligence of the role of personal factors (particularly PYD attributes), studies on the role of PYD qualities in these two areas of adolescent academic well-being are very important. Besides, the related findings would shed light not only on research but also on intervention programs that may promote academic well-being among high school students.

The findings support Hypothesis 1a, showing that PYD qualities at Wave 1 positively predicted the participants’ satisfaction with their academic study curriculum at Wave 6. Theoretically, as a positively developed adolescent would possess high competence in different domains and develop a high self-efficacy (Catalano et al., 2004; Lerner, 2005), he or she would feel more capable and have better planning, self-management, and self-regulation in academic study. A positively developing adolescent would also gain more social support from peers and teachers in their academic study, and they would have higher spirituality and resilience in facing difficulties. The present study is pioneering in that it provides direct evidence of the positive influence of PYD on adolescents’ academic satisfaction. The findings are consistent with some isolated research findings that PYD indicators positively predicted academic satisfaction and school satisfaction (Lent et al., 2007; Sun, 2016; Urquijo and Extremera, 2017). This study expanded the empirical evidence of the value of PYD development, showing that PYD qualities would provide positive influence on adolescents in different aspects, including their satisfaction with their academic studies.

The results also support Hypothesis 1b, revealing that PYD at Wave 1 negatively predicted perceived academic stress at Wave 6. The finding is consistent with a few studies showing that some personal strength factors negatively predicted perceived academic stress. For example, a study showed that perceived stress was predicted by the ability of emotional regulation and management (Bao et al., 2015), which is one important indicator of PYD. Another study revealed that character strength such as grit negatively predicted perceived stress level among college students (Lee, 2017), which strongly suggests that stress could be interpreted as “the subjective psychological appraisal of an event as threatening or not” and “psychological resources may influence one’s appraisal of an event” (Lee, 2017, 148–149). Positive youth development qualities such as psychosocial competence, confidence, character strengths, and important connections (Catalano et al., 2004; Lerner, 2005) are essential psychological resources for adolescents. Adolescents with higher levels of PYD qualities would possess more psychological resilience to appraise a negative or threatening event more positively, which leads to reduced stress in different domains, including academic stress.

The results support Hypothesis 2a, showing that PYD (Wave 1) positively predicted LS (Wave 3). This is in line with the literature that psychological well-being and different indicators of PYD positively predicted their life satisfaction: one study showed that social support and self-esteem were important antecedents of satisfaction with life (Chen et al., 2017); Howell et al. (2013) found that meaning in life (a PYD quality) positively predicted satisfaction with life; Rey et al. (2011) revealed that both emotional intelligence and self-esteem (PYD qualities) positively predicted satisfaction with life. As existing studies commonly focused on one to two PYD qualities in a single study, the present study’s focus on different dimensions of PYD qualities and adoption of longitudinal research method contributes to the existing literature by suggesting that PYD qualities are important precursor of satisfaction with life.

The results also support Hypothesis 2b, showing that satisfaction with life at Wave 3 positively predicted students’ satisfaction with their academic study programs at Wave 6. The findings support the “top-down model” that global life satisfaction is a relatively stable psychological strength which would influence an individual’s perceptions of specific aspects of life or experiences (Diener, 1984; Scherpenzeel and Saris, 1996). While no study has been done to examine the relationship between global satisfaction with life and specific satisfaction with academic study, the results are consistent with some existing studies supporting the “top-down model” that global satisfaction with life would influence satisfaction with specific life aspects such as job satisfaction, career satisfaction, and relationship satisfaction (Judge and Watanabe, 1993; Diener and Tay, 2017). Hence, the present study constitutes a theoretical advance in this area.

Hypothesis 2c was also supported by the present study which showed that life satisfaction (Wave 3) negatively predicted academic stress (Wave 6). This finding strengthens the existing literature suggesting that life satisfaction could be a precursor or antecedent of perceived stress (e.g., Saleh et al., 2017; Tang and Chan, 2017). Theoretically, as “a global propensity to experience things in positive ways” (Diener, 1984, p.565), higher life satisfaction would enable an individual to appraise an environmental stressor, such as that of academic studies, as less negative and devastating as well as to cope more positively, which would eventually lead to a lower level of stress. This conjecture constitutes an exciting area for future research.

Finally, the results of the present study support Hypotheses 3a and 3b, showing that life satisfaction partially mediated the positive influence of PYD on academic satisfaction but fully mediated the negative influence of PYD on academic stress. The findings suggest that global life satisfaction is an important mechanism underlying the relationship between PYD and academic well-being, where a higher level of PYD would lead to a higher level of satisfaction with academic study but a lower level of perceived stress. The results are significant as very few studies have investigated the mediating function of global satisfaction with life on the relationship between psychological well-being such as PYD qualities and adolescent academic well-being. Particularly, the extant literature on the mediation role of satisfaction with life focused mainly on satisfaction with life being an underlying mechanism in the relationship between environmental antecedents (e.g., stressful life events and authoritative parenting) and adolescents’ problem behaviors such as internalizing problems (e.g., McKnight et al., 2002; Suldo and Huebner, 2004). Only few studies revealed that life satisfaction mediated the impact of PYD on adolescent problem behavior (Sun and Shek, 2010, 2012, 2013). Therefore, the results of this study expand the existing literature by highlighting the important role of satisfaction with life in the relationship between PYD and adolescents’ academic well-being.

This study has important practical implications. It provides school educators with important knowledge about the important role of PYD and life satisfaction in adolescent academic well-being. Therefore, school educators and administrators could try to promote their students’ PYD and life satisfaction through different measures or interventions. One possibility is to implement PYD programs to promote life satisfaction of the program participants. In the Chinese context, the Project P.A.T.H.S. has been shown to promote life satisfaction in Chinese high school students in Hong Kong (Ma et al., 2019a,b) and China (Zhu and Shek, 2020). The development and implementation of educational and intervention programs to promote students’ PYD qualities and life satisfaction would be very meaningful for helping educators and school administrators to reduce the academic stress of students and elevate their academic satisfaction.

Despite the pioneering nature of the study in the field of adolescent life satisfaction and academic well-being, this study has limitations. First, this study only tested the predictive role of PYD qualities in adolescent academic well-being indexed by academic satisfaction and academic stress, and the mediating effect of life satisfaction on the relationship. Because the literature also suggests the predictive role of stress and domain-specific satisfaction in life satisfaction, alternative models should be tested in future research to further advance the understanding of the relationships among the factors under study. Second, despite having a large sample size, this study was mainly based on Hong Kong secondary school students. Studies on adolescents in other Chinese cultures and non-Chinese cultures should also be conducted to replicate the results. Third, it is also interesting to examine whether the mediation effects and the relationships among variables are the same for boys and girls. Future research is needed in this direction. Fourth, some subscales of CPYDS had relatively lower reliability, which might be due to the fewer item numbers, although the “mean inter-item correlation” and “mean item-total correlation” values are acceptable. Future research could be conducted by using the original version of CPYDS to retest the mediation model. Despite these limitations, this study is pioneering in that it examines the longitudinal predictive role of PYD qualities in academic satisfaction and stress and in uncovering the underlying mechanism played by life satisfaction.

## Data Availability Statement

The datasets generated for this study are available on request to the corresponding author.

## Ethics Statement

This study was approved by the Human Subjects Ethics Sub-Committee (HSESC) (or its Delegate) of The Hong Kong Polytechnic University. Formal written consent was obtained from all participating schools, students, and their parents.

## Author Contributions

DS conceived of the research, contributed to all stages of the research work, and critically revised the different versions of the manuscript drafted by WC. WC conducted data analyses, drafted the manuscript, and revised the manuscript based on the comments provided by DS.

## Conflict of Interest

The authors declare that the research was conducted in the absence of any commercial or financial relationships that could be construed as a potential conflict of interest.
